# Correction: Construction of a homologous series of metal nanoclusters and implications for structure–activity correlations

**DOI:** 10.1039/d5sc90229k

**Published:** 2025-10-23

**Authors:** Qinzhen Li, Tingting Jiang, Sha Yang, Jinsong Chai, Haizhu Yu, Manzhou Zhu

**Affiliations:** a School of Materials Science and Engineering, Anhui University Hefei Anhui 230601 China chaijs@ahu.edu.cn; b Department of Chemistry and Centre for Atomic Engineering of Advanced Materials, Key Laboratory of Structure and Functional Regulation of Hybrid Materials of Ministry of Education, Institutes of Physical Science and Information Technology, Anhui Province Key Laboratory of Chemistry for Inorganic/Organic Hybrid Functionalized Materials, Anhui University Hefei Anhui 230601 China yuhaizhu@ahu.edu.cn zmz@ahu.edu.cn

## Abstract

Correction for ‘Construction of a homologous series of metal nanoclusters and implications for structure–activity correlations’ by Qinzhen Li *et al.*, *Chem. Sci.*, 2025, https://doi.org/10.1039/d5sc06282a.

The authors regret that an incorrect CCDC number was included in the original article. CCDC number 2341165 should be replaced with 2374165. The correct Supplementary Information document and cif file have been updated on the original article.

The Royal Society of Chemistry apologises for these errors and any consequent inconvenience to authors and readers.

